# Hydrogels for Cell Delivery

**DOI:** 10.3390/gels4030058

**Published:** 2018-07-02

**Authors:** Esmaiel Jabbari

**Affiliations:** Biomimetic Materials and Tissue Engineering Laboratory, University of South Carolina, Columbia, SC 29208, USA; jabbari@cec.sc.edu; Tel.: +01-803-777-8022

Hydrogels have a three-dimensional crosslinked molecular structure which absorb large quantities of water and swell in a physiological environment. Hydrogels are a class of polymers made from hydrophilic repeat units that interact with water molecules by hydrogen bonding, polar and ionic interaction to take up water many times the initial polymer weight. Further, the polymer chains in the hydrogel are linked via crosslinks to form an infinite network to prevent dissolution of the polymer chains in an aqueous medium. Hydrogels can be natural or synthetic. Due to their high water content, oxygen molecules, nutrients, peptides, proteins, ribonucleic acid (RNA) and deoxyribonucleic acid (DNA) biomolecules diffuse readily through hydrogels. Further, cells immobilized in hydrogels maintain their viability and function. As a result of these benefits, hydrogels are used extensively in medical applications for replacement, repair, and regeneration of soft biological tissues. There are >8000 references to hydrogels in PubMed and >15,000 in Web of Science search engines.

Recently, hydrogels have been used as a matrix for delivery of cells and morphogens to the site of injury in regenerative medicine. Natural as well as synthetic hydrogels are used in tissue replacement, repair, and regeneration. Natural hydrogels can be derived from plants or animals. Plant-derived hydrogels include polysaccharide-based agarose, alginate, and carboxymethyl cellulose. Animal-derived hydrogels include polysaccharide-based, such as hyaluronic acid, and protein-based, such as collagen, gelatin, chitosan, and fibrin. In particular, injectable and in-situ hardening hydrogels functionalized with photocrosslinkable moieties are very attractive for repairing or regenerating irregularly-shaped tissue injuries using minimally-invasive arthroscopic procedures. In that approach, a suspension of therapeutic cells, morphogens, and growth factors in a functionalized hydrogel precursor solution is injected through a catheter to the injury site guided by imaging. After injection, the precursor solution is hardened or gelled by shinning ultraviolet or visible light enabled catheter.

More recently, hydrogels are being used as bioinks for printing cells, morphogens, and growth factors such that the spatial organization of the printed cells and growth factors mimic that of the target tissue. The hydrogel ink in these cellular constructs serves as an extracellular glue to maintain dimensional ability and provide mechanical strength to the construct. The hydrogel also provides ligands for specific interactions between the cell surface receptors and the extracellular matrix (ECM) guide cellular events like adhesion, migration, mitosis, differentiation, maturation, and protein expression. Multiple printing heads are used to print tissue constructs with many cell types and growth factors.

The articles in this Special Issue provide exemplary reviews and research works related to the use of hydrogels in tissue engineering and regenerative medicine. Although cells encapsulated in hydrogels maintain their viability and function, the high water content significantly reduces the hydrogel’s mechanical strength. As a result, hydrogels unaided cannot be used a matrix for regeneration of load-bearing tissues such as bone. To mitigate this issue, Kumar and collaborators describe in their article titled “A Bioactive Hydrogel and 3D Printed Polycaprolactone System for Bone Tissue Engineering” the development of a novel hard–soft biphasic construct with a gyroid geometry by 3D printing. In this approach, a stiff poly(ε-caprolactone) (PCL) polymer was used to print the hard phase of the construct in a gyroid geometry, whereas a combination of alginate and gelatin was used to print the soft phase as a carrier for osteoblast progenitor cells. The gyroid geometry of the hard phase increased the volume of the soft phase which, in turn, increased cell loading and the extent of osteogenesis.

A major complication of cellular tissue constructs is microbial fouling after implantation. Individually there are viable options for sterilization of biomaterials, growth factors, and cells. However, complete sterilization of cells, growth factors, and biomaterials collectively in a tissue construct is complicated, even with the use of anti-bacterial and anti-fungal agents. Therefore, strategies that can reduce microbial fouling can significantly enhance their suitability in clinical applications. In that regard, Yu and collaborators describe in their review titled “Polyampholyte Hydrogels in Biomedical Applications” the properties of polyampholyte hydrogels and their non-fouling characteristics. Polyampholytes are an interesting class of hydrogels that possess both positive and negatively charged units in their structure. The interaction between the positive and negatively charged units imparts anti-fouling properties to the hydrogel which can be exploited in tissue engineering applications.

Natural hydrogels are widely used as a carrier for cells in tissue engineering because they contain sequences of amino acids that interact with cell surface receptors to guide cell function and expression. However, it is difficult to tailor the multitude of ligand–receptor interactions in natural matrices to a particular application in regenerative medicine. Further, natural hydrogels suffer from batch-to-batch variability in composition, limited thermal and mechanical stability, and relatively fast and uncontrolled enzymatic degradation. Conversely, synthetic hydrogels have tunable physical and mechanical properties for a wide range of applications in medicine, but they lack instructive interactions with the encapsulated cells. Therefore, there is a need to develop novel synthetic approaches to modify hydrogels with cell-adhesive ligands. Cipolla, Russo and collaborators in “Bioresponsive Hydrogels: Chemical Strategies and Perspectives in Tissue Engineering” and Varghese and collaborators in “Hydrogels as Extracellular Matrix Analogs” describe strategies and approaches to produce functional, cell-responsive hydrogels for applications in regenerative medicine.

In regenerative medicine, a mixture of growth factors as well as many ligand receptor interactions, physical and mechanical factors are involved in differentiation and maturation of progenitor cells to a specific lineage. Therefore, there is a need to develop high-throughput techniques to screen for these factors within a 3D tissue culture system. The review by Dr. Smith Callahan titled “Combinatorial Method/High Throughput Strategies for Hydrogel Optimization in Tissue Engineering Applications” highlights the strengths and disadvantages of design of experiment, arrays and continuous gradients and fabrication challenges for hydrogel optimization in tissue engineering applications.

The interaction of receptors on the cell surface with ECM ligands starts a cascade of signaling from the cell membrane to the cell cytoplasm and the nucleus to activate/deactivate genes of interest. The gene activation in turn leads to protein expression and secretion of the desired ECM and tissue regeneration. Although cell–ECM interactions have been extensively studied in 2D culture system, more work is needed to understand signal transduction in biomimetic 3D cultures with cells encapsulated in hydrogels. Ventre and Netti in “Controlling Cell Functions and Fate with Surfaces and Hydrogels: The Role of Material Features in Cell Adhesion and Signal Transduction” review signal transduction for cells in hydrogels that captures features of the natural cellular environment, such as dimensionality, remodeling and matrix turnover. 

